# Predictive Value for Increased Red Blood Cell Distribution Width in Unprovoked Acute Venous Thromboembolism at the Emergency Department

**DOI:** 10.1177/10760296231193397

**Published:** 2023-09-10

**Authors:** Cláudia Febra, Verónica Spinu, Filipa Ferreira, Victor Gil, Rui Maio, Deborah Penque, Ana Macedo

**Affiliations:** 1 Department of Intensive Care, 199396 Hospital da Luz Lisboa, Lisbon, Portugal; 2 Faculty of Medicine, University of Porto, Porto, Portugal; 3 Department of Emergency Medicine, 467035 Hospital Beatriz Angelo, Loures, Portugal; 4 Center of Cardiovascular Risk and Thrombosis, Hospital da Luz Torres de Lisboa, Lisbon, Portugal; 5 Department of General Surgery, 199396 Hospital da Luz Lisboa, Lisbon, Portugal; 6 59020 Laboratory of Proteomics, Department of Human Genetics, Instituto Nacional de Saúde Dr Ricardo Jorge, Lisbon, Portugal; 7 Algarve Biomedical Center (ABC), Faro, Portugal; 8 615668 Faculty of Medicine and Biomedical Sciences (FMCB), University of Algarve, Faro, Portugal

**Keywords:** red cell distribution width, erythrocytes indices, venous thromboembolism

## Abstract

Acute venous thromboembolism (VTE) is a common worldwide disease admitted to emergency departments (ED), usually presenting as pulmonary embolism or lower limb deep vein thrombosis (DVT). Due to the lack of typical clinical and biomarker diagnostic features of unprovoked VTE, early identification is challenging and has direct consequences on correct treatment delay. Longitudinal, prospective, observational study. Patients admitted to ED with a suspicion of unprovoked acute VTE between October 2020 and January 2021 were included. Clinical and laboratorial variables were compared between VTE positive and negative diagnoses. Red cell distribution width (RDW) cut point was determinate through a receiver operating characteristic analysis. RDW accuracy, sensitivity, and specificity were calculated. Fifty-eight patients were analyzed. And 82.8% of suspected patients with VTE were diagnosed with an acute thrombotic event confirmed by imaging examination. In patients with VTE, RDW at admission in ED was higher than with other diagnosis, respectively, 14.3% (13.2-15.1) and 13.5% (13.0-13.8). Platelet count was the only additional characteristic that revealed difference between the 2 groups (264×10^9^/L for VTE and 209×10^9^/L for non-VTE). Logistic regression models showed good discriminatory values for RDW≥14%, with an area under the curve (AUC) = 0.685 (95% confidence interval, 0.535-0.834). These findings were more pronounced in isolated DVT, with a sensitivity of 76.9%, specificity 100%, and accuracy 85.7%. Our study demonstrated a significant association between an early high RDW and the diagnosis of acute unprovoked DVT. RDW ≥ 14% has an independent predictor of unprovoked VTE in adult patients.

## Introduction

Pulmonary embolism (PE) and lower limb deep vein thrombosis (DVT) are the most frequent clinical expressions of acute venous thromboembolism (VTE), resulting in an annual incidence equal to that of stroke.^1^ Acute VTE episodes have a significant and constant impact on health resources, accounting for 1 million emergency visits in the United States, being half of them admitted to hospital.^2^ VTE episodes occurring out-of-hospital, compared to those acquired in hospitals, are about 40 times less frequent but have a wide range of related risk factors and morbidities.^3^ Due to the heterogeneity of these demographic and clinical characteristics, particularly in PE cases, and the absence of specific and sensitive biomarkers, VTE diagnosis in the emergency departments (ED) is a high challenge to physicians. No single clinical symptoms are predictive or exclude PE, although the lack of dyspnea slightly reduces the probability of PE.^4^ The clinical presentation of VTE is unspecific,^5^ particularly for unprovoked cases, characterized by the absence of any transient or persistent environmental risk factor.^6^ When patients are admitted to the ED, the risk of VTE is assessed by clinical gestalt or clinical scores according to symptoms and existing risk factors.^7^ This risk assessment is the first diagnostic step after suspicion of VTE and it is usually followed by D-dimer measurements to determine whether further investigations are required. Small or intermediate clinical probabilities and negative D-dimers can exclude PE^8‐10^ and DVT,^11^ but the results of positive D-dimers have a low-positive predictive value and are not useful for VTE diagnosis.^12‐14^ The key to diagnosis of PE and DVT is still to perform imaging examinations, computed tomography (CT) angiogram, and Doppler ultrasound, respectively. Accessibility, radiation exposure, time-to-exam and high costs are some of the critical aspects that are due to the need of such examinations. The absence of alternative diagnostic biomarkers for VTE has a direct impact on the percentage of 60% undiagnosed cases among all VTE-attributable deaths.^15^

Efforts have been made to increase the knowledge on the diagnostic and prognostic importance of red cell indices of cardiovascular diseases. Red blood cell distribution width (RDW) is a measurement of size heterogeneity of circulating erythrocytes,^16^ which has been studied as a prediction tool for cardiovascular and thrombotic diseases, including PE,^17‐19^ although some conflicting results have prevented it from being used in everyday practice. The diagnostic role of RDW for acute VTE is even less well understood. A previous retrospective study on VTE found an association between high RDW at admission to ED and acute VTE, and determined a cutoff of 14.6%, with an Negative Predictive Value (NPV) of 0.85 for DVT and 0.91 for PE, suggesting that RDW could supplement clinical preliminary examinations to exclude acute VTE attacks.^20^ This study, even considering some limitations, has set a possible threshold of RDW > 14% at admission for distinguishing VTE from non-VTE patients in acute care setting.

In response to the same questions, we decided to investigate the early RDW values in patients admitted to the ED for suspected acute unprovoked VTE episodes and compare clinical characteristics and biomarkers of patients with confirmed and excluded VTE diagnosis.

## Methods

### Study Design and Settings

We conducted a prospective longitudinal, observational study aiming to describe and compare clinical and laboratory parameters of patients admitted in an ED with suspected VTE, including PE and DVT, from October 2020 to January 2021.

### Participants

The study included patients admitted in the ED who were clinically suspected of an acute VTE and referred, according to usual clinical practice, for imaging examinations for diagnosis confirmation. Inclusion criteria were individuals aged ≥18 years with unprovoked suspected acute VTE cases (DVT or PE) admitted in the ED, or transferred to the ED (eg, from an outpatient clinic or other hospital) with a diagnosis of suspected acute VTE <24 h. Patients were excluded if there was VTE suspicion more than 24 h before admission to the ED, life expectancy of <3 months, active neoplasia, pregnancy or breastfeeding, COVID-19 diagnosis, and fever at admission.

### Ethical Considerations

The study was conducted in accordance with current International Council for Harmonisation Good Clinical Practice guidelines, and according to Declaration of Helsinki principles. The study protocol was approved by Hospital Beatriz Angelo Ethical Committee and Board. Written informed consent was obtained from all enrolled subjects.

### Procedures and Data Collection

Data were collected from patients’ medical records. All patients were screened for the presence of VTE and depending on the clinical suspicion thoracic angiotomography for PE and/or lower limb venous Doppler ultrasound for DVT were performed according to usual clinical practice.

The VTE diagnosis was ruled out, if the imaging examinations were negative for the initial suspicion, and another diagnosis was established.

### Clinical, Biochemical Parameters, and Imaging Examinations

Collected data included: demographic data (sex and age), present and past medical history, previous medication, physical examination, vital signs, peripheral oxygen saturation, and laboratory parameters such as red blood cells, hemoglobin, hematocrit, RDW, platelets, total leukocyte count, creatinine, D-dimer, and C-reactive protein. All patients performed an imaging examination in the first 24 h after admission to the ED, according to physician's dispositions. The imaging considered to include patients in the analysis consisted of thoracic CT angiography for PE-suspected cases and lower limb Doppler ultrasound for DVT suspicions. Patients were allowed to perform both examinations in the same period.

### Statistical Analysis

Continuous data were presented as median and interquartile ranges, mean and standard deviation (SD), maximum and minimum. Discreet variables were presented as absolute and relative frequencies. The normality of the data was assessed using the Shapiro-Wilk test.

Between-group (VTE vs non-VTE) comparisons of continuous variables were performed using Mann-Whitney U tests. Categorical variables were compared using Fisher's exact test.

A receiver operating characteristic curve analysis was used to evaluate the diagnostic value and to define the diagnostic cutoff value of RDW concentrations. Using the cutoff point defined, the RDW were compared between VTE versus non-VTE by the Fisher's exact test.

A logistic regression model was implemented to interrogate the significance of the association of RDW in patients with VTE, considering demographic and other clinical variables.

A significant level of 0.05 was considered for all the analysis.

SPSS 26.0 statistical software was used to analyze the data.

## Results

### Patients’ Characteristics and VTE Diagnosis

A total of 59 participants were initially enrolled. One patient abandoned ED without any imaging examination and was excluded. Of the 58 patients included in the analysis, 44 completed thoracic CT angiography and 33 lower limb Doppler ultrasound with diagnostic results of adequate quality, with 19 patients carrying out both examinations. The characteristics of 58 patients are summarized in Table 1. There was a total of 48 patients (82.8%) with an acute VTE diagnosis and the remaining 10 patients received an alternative diagnosis. There was no significant difference between patients with VTE and without VTE diagnosis in terms of clinical presentation. Considering 44 patients who completed a thoracic CT angiography, 35 (80%) presented features of acute PE, as shown in Table 2. Of those patients who performed a lower limb Doppler ultrasound, 70% (25 cases) presented were diagnosed with acute DVT. According to the imaging examinations, 21% (10 cases) of patients with VTE were diagnosed with both acute PE and acute DVT.

**Table 1.** Study Participants’ Baseline Characteristics.

Characteristic	All patients	Patients with VTE	Patients without VTE	*P* value*
Age, y, median (IQR), maximum-minimum (N)	65.0 (44.5-84.5)	64.5 (42.8-83.8)	72.5 (55.8-88.0)	.415
	19-90 (N = 58)	19-89 (N = 48)	27-90 (N = 10)	
Female, N (%)	38 (65.5)	31 (64.6)	7 (70.0)	1.0#
	(N = 58)	(N = 48)	(N = 10)	
Standing heart rate at rest, bpm, mean ± SD	88.0 (76-102)	88.0 (76-103)	91.0 (82-101)	.597
	50-130 (N = 48)	50-130 (N = 41)	53-127 (N = 7)	
Oxygen saturation (SpO_2_), %, mean ± SD	97.0 (95-98)	97.0 (95-98)	96.0 (96-98)	.618
	85-100 (N = 46)	85-100 (N = 39)	96-98 (N = 7)	
Hemoglobin, g/dL	13.4 (12.3-14.5)	13.4 (12.5-14.5)	13.9 (11.6-14.1)	.597
	7.3-15.8 (N = 48)	7.3-15.8 (N = 41)	11.2-15.3 (N = 7)	
Hematocrit, %	42.2 (36.9-44.3)	42.4 (37.2-44.3)	39.8 (36.2-42.7)	.683
	24.6-47.4 (N = 48)	24.6-47.4 (N = 41)	33.9-45.8 (N = 7)	
RDW, %	14 (13.1-14.7)	14.3 (13.2-15.1)	13.5 (13.0-13.8)	.004
	11.4-25.4 (N = 48)	11.8-25.4 (N = 41)	11.4-13.9 (N = 7)	
Total leukocyte count, 10^12^/L	10.2 (7.6-11.6)	10.08 (7.5-11.4)	10.32 (7.8-13.5)	.683
	4.2-18.8(N = 48)	4.2-18.8 (N = 41)	7.3-15.2 (N = 7)	
Neutrophil %	69.6 (63.4-76.7)	69.8 (63.5-76.5)	69.0 (57.4-81.6)	.683
	37.6-88.3 (N = 48)	37.6-88.3 (N = 41)	44.4-82.2 (N = 7)	
Lymphocyte %	19.9 (13.7-25.4)	19.6 (14.2-25.0)	20.2 (10.4-32.6)	.683
	2.9-50.7 (N = 48)	2.9-50.7 (N = 41)	10.3-43.4 (N = 7)	
Platelet count, 10^9^/L	256.5 (210-354)	264.0 (212.5-355)	209.0 (190-230)	.041
	167-791 (N = 48)	174-791 (N = 41)	167-441 (N = 7)	
C-reactive protein, mg/dL	2.6 (0.8-5.7)	2.3 (0.8-4.7)	9.5 (1.4-19.5)	.346
	0.2-40.2 (N = 43)	0.2-40.2 (N = 37)	0.4-22.6 (N = 6)	
D-dimer, μg/mL	6.9 (3.3-10.8)	7.2 (3.8-11.3)	2.2 (0.9-10.8)	.413
	0.9-32.5 (N = 41)	1.3-32.5 (N = 35)	0.9-16.2 (N = 6)	
Creatinine	0.9 (0.8-1.8)	0.9 (0.8-1.1)	1.0 (0.8-1.8)	.867
	0.7-2.3 (N = 47)	0.6-1.8 (N = 41)	0.7-2.3 (N = 7)	

Abbreviations: IQR, interquartile range; RDW, red cell distribution width; VTE, venous thromboembolism.

* Mann-Whitney U test.

# Fisher’s exact test.

**Table 2.** Results of Imaging Examinations for Location of VTE (N = 48) and Diagnosis of Non-VTE (N = 10).

Location of disease or diagnosis	Number of patients
PE	35
Main pulmonary artery trunk	4
Bilateral multiple segmental	18
Unilateral multiple segmental	6
Segmental single	3
Subsegmental	5
DVT	25
Proximal	18
Distal	7
No VTE	10
Erysipelas	4
Superficial phlebitis	3
Anxiety	1
Pericardial effusion	1
Tachycardia	1

Abbreviations: DVT, deep vein thrombosis; PE, pulmonary embolism; VTE, venous thromboembolism.

In terms of laboratory investigation, there were significant differences on platelet count and RDW between VTE and non-VTE patients. There were no differences in hemoglobin, hematocrit, leukocytes, and D-dimer. Since RDW was the variable showing the strongest association with acute VTE in the univariate analysis, we focused on RDW for further analyses.

All patients were discharged from the hospital.

### Diagnostic Performance of RDW *≥* 14%

**Figure 1.**
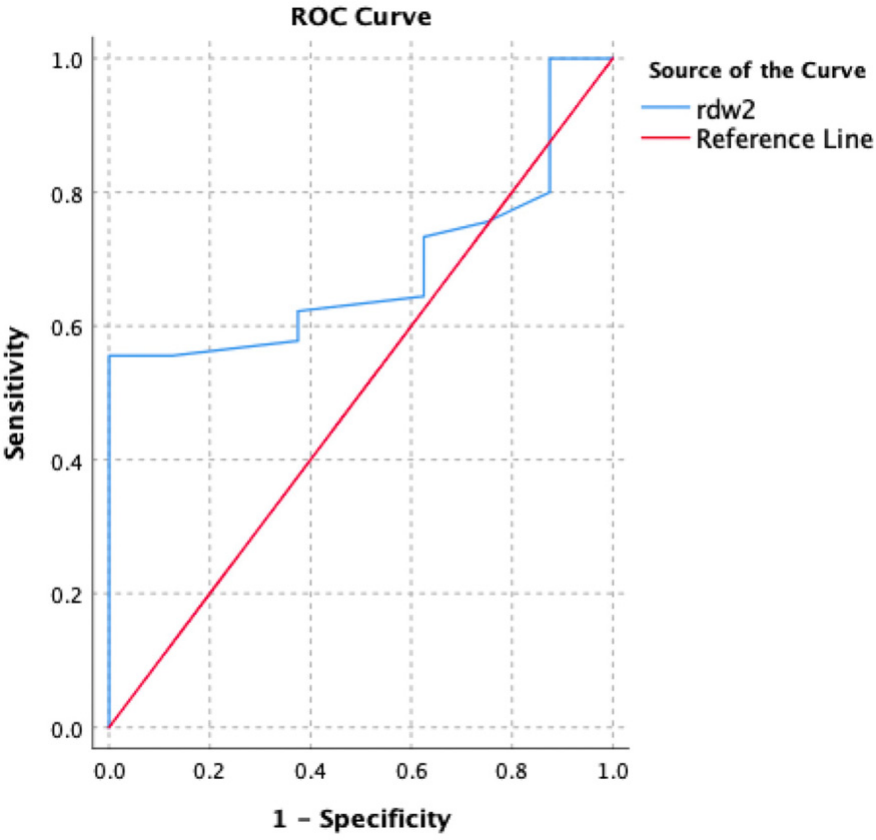
ROC analysis for RDW. Abbreviations: RDW, red cell distribution width; ROC, receiver operating characteristic.

The diagnostic accuracy of RDW ≥ 14% for determining an acute VTE, as quantified by the area under the curve, was 0.685 (95% CI, 0.535-0.834; Figure 1). At a threshold of ≥14%, RDW ruled in 47% of individuals, with a sensitivity of 55.6%, specificity 100%, and accuracy 79.0% (95% CI, 65.6%-89.0%). For isolated PE RDW ≥ 14% had a sensitivity of 40.9% and the specificity of 100%, with an accuracy of 56.7%. And, for isolated DVT RDW ≥ 14% had a sensitivity of 76.9%, specificity 100%, and accuracy 85.7% (Table 3).

**Table 3.** RDW ≥ 14% Sensitivity, Positive Predictive Value, Specificity, Negative Predictive Value, and Accuracy.

	VTE	PE	DVT
Sensitivity (%; 95% CI)	55.6	40.9	76.9
	40.0-70.4	20.7-63.7	46.2-94.9
Positive predictive value (%; 95% CI)	100	100	100
	86.3-100	66.3-100	69.2-100
Specificity (%; 95% CI)	100	100	100
	63.1-100	63.1-100	63.1-100
Negative predictive value (%; 95% CI)	71.6	38.1	72.7
	51.5-86.9	18.1-61.6	39.0-94.0
Accuracy (%; 95% CI)	79.0	56.7	85.7
	65.689.0	37.5-74.6	63.7-97.0

Abbreviations: CI, confidence interval; DVT, deep vein thrombosis; PE, pulmonary embolism; RDW, red cell distribution width; VTE, venous thromboembolism.

Logistic regression analysis confirmed RDW as a predictor of VTE, being RDW > 14% and age the only independent predictors of acute VTE.

## Discussion

Our study aims to improve the early, rapid, and accurate diagnosis of patients who present to the ED with a suspicion of acute VTE. We evaluated the diagnostic value of clinical and laboratory findings at the time of ED presentation, keeping in mind the need for urgent recognition of acute VTE in this setting, for the emergent initiation of anticoagulation and, in the catastrophic situations, fibrinolytics therapy.^21‐23^ Our findings showed that RDW value and platelet count were the only variables with a significant association with acute DVT. A high RDW (≥ 14.0%) at admission to ED was significantly associated with the presence of acute DVT, independent of other clinical and laboratory variables, including D-dimer. These findings are consistent with those of previous general population studies or retrospective series,^24‐30^ that utilized healthy controls for comparison. RDW was demonstrated to be higher than normal cutoffs in such diverse conditions as cardiac stent thrombosis,^31^ heart failure,^32^ inflammatory diseases,^33^ or acute exacerbation of chronic obstructive pulmonary disease.^34^ One of the studies^24^ described the association of an RDW > 14% to acute deep and superficial vein thrombosis at an ED at the admission, but it mixed unprovoked and provoked VTE—by cancer, trauma, infection, surgery, and other causes—which can be very different populations in terms of RDW. More so, it used outpatients on routine follow ups as controls, expected to present normal RDW, thus not completely reliable as a comparator if we consider that RDW is higher in many of the diseases presenting to the ED. To the best of our knowledge, this is the first study to document that high RDW is associated with the presence of VTE among acutely ill patients who present clinical features suspicious of acute unprovoked VTE, thus distinguishing confirmed from excluded VTE cases. The detailed analysis of RDW > 14% confirmed its high specificity and positive predictive value for distinguishing between acute VTE and other alternative acute diseases, in line with the aforementioned results. We could also observe that all patients admitted to the ED who were diagnosed non-VTE conditions had higher than normal RDW measurements, but RDW value was always inferior to 14%. In the light of these data, we propose that RDW > 14% could be tested as an early biomarker to predict acute VTE among acutely ill patients.

For unprovoked VTE attacks, which make up to a half of all acute VTE episodes^35^ and present a much higher risk of recurrence compared to patients with VTE provoked by a major transient risk factor,^36,37^ baseline risk assessment of patients with suspected VTE on ED admission may be particularly difficult. The lack of typical clinical characteristics, which our study also found, increases the risk of unprovoked VTE being missed. The use of a high D-dimer should be discouraged to differentiate acute VTE in situations of acute medical or surgical illnesses, even with cutoff adjustments.^38‐40^ Considering the high prevalence of high D-dimer values in these circumstances, ordering costly, often unavailable, and potentially harmful, contrasted imaging tests for exclusion of VTE is necessary. Our study found that an RDW > 14% alone, for both PE and DVT, had a significant positive predictive value, which is comparable to the association of clinical assessment and D-dimer for low-risk patients. We have arguments to suggest that this diagnostic parameter should be further studied, along with clinical characteristics and risk factors, to augment both the specificity and sensitivity of scores in the early diagnosis of acute VTE. The low specificity demonstrated by D-dimer for both PE and DVT,^12,41^ and that contrasts with its significant negative predictive value, limits its usefulness among acute ED patients. By contrary, further evidence on a high positive predictive value of RDW > 14% in the first hours of acute VTE, particularly in DVT that showed more consistent results when we analyzed separate subgroups of venous thrombosis, may add some value to the existing algorithms. In fact, the combination of both D-dimer and RDW measurements may, eventually, contribute to a higher predictive value of clinical and easily ready blood tests to the diagnosis before imaging examinations. Being this confirmed, we could expect a strong impact in clinical settings where imaging examinations are not immediately accessible and the decision making for beginning anticoagulation is needed.

Our study had several limitations. The first one is directly related to COVID-19 pandemics. During COVID-19 pandemics, worldwide ED were re-arranged to handle patients with potential or confirmed SARS-CoV-2. There was a modification of accessibility to EDs during COVID-19 pandemics, overloaded with COVID-19 cases that consumed most healthcare resources, preventing patients from receiving clinical care. ED visits declined 44%,^42^ and clinical assessment of all incoming patients were likely altered from the “usual” procedures. Furthermore, disorders like PE and DVT, which require extensive workups, may have been significantly affected by the reduction in one-third of the diagnostic examinations. Although in Portugal 48% reduction in ED visits did not impact the mix case of visiting patient,^43^ little is known about the use of diagnostic imaging tests in the ED. In the light of these details, we could advise against extrapolating the findings to a population not affected by the COVID-19 pandemic. The same caution should be applied due to our small-sized and single-center population. Although we collected data on concomitant diagnosis (ICD-10), we were not exhaustive and lack data on nutritional status, shown to modify RDW among unhealthy populations.^20^ Finally, we would like to address the dearth of information regarding patient outcomes. Aside from the mortality outcome, we lacked information on the clinical evolution during the in-hospital stay and after hospital discharge. The analysis of those variables could provide some insight into the potential prognostic significance of RDW over the course of the illness.

In summary, we have demonstrated that high RDW and platelet count, 2 parameters that are inexpensive and easily obtainable, were independently associated with an increased risk of venous thrombosis. Further research is necessary to determine whether this association is causative. Future studies should target the evaluation of these variables as predictors of a recurrent event, which could assist in future decisions regarding anticoagulation and prophylaxis.

## References

[1] Heit JA Spencer FA White RH . The epidemiology of venous thromboembolism. J Thromb Thrombolysis. 2016;41(1):3‐14. doi:10.1007/s11239-015-1311-6 26780736 PMC4715842

[2] Singer AJ Thode HC Peacock WF . Admission rates for emergency department patients with venous thromboembolism and estimation of the proportion of low risk pulmonary embolism patients: a US perspective. Clin Exp Emerg Med. 2016;3(3):126‐131. doi:10.15441/ceem.15.096 27752630 PMC5065336

[3] Heit JA . Epidemiology of venous thromboembolism. Nat Rev Cardiol. 2015;12(8):464‐474. doi:10.1038/nrcardio.2015.83 26076949 PMC4624298

[4] West J Goodacre S Sampson F . The value of clinical features in the diagnosis of acute pulmonary embolism: systematic review and meta-analysis. QJM. 2007;100(12):763‐769. doi:10.1093/qjmed/hcm113 18089542

[5] Kruger PC Eikelboom JW Douketis JD Hankey GJ . Deep vein thrombosis: update on diagnosis and management. Med J Aust. 2019;210(11):516‐524. doi:10.5694/mja2.50201 31155730

[6] Kearon C Ageno W Cannegieter SC , et al. Categorization of patients as having provoked or unprovoked venous thromboembolism: guidance from the SSC of ISTH. J Thromb Haemost. 2016;14(7):1480‐1483. doi:10.1111/jth.13336 27428935

[7] Konstantinides SV Meyer G Becattini C , et al. 2019 ESC guidelines for the diagnosis and management of acute pulmonary embolism developed in collaboration with the European Respiratory Society (ERS). Eur Heart J. 2020;41(4):543‐603. doi:10.1093/eurheartj/ehz405 31504429

[8] Carrier M Righini M Djurabi RK , et al. VIDAS D-dimer in combination with clinical pre-test probability to rule out pulmonary embolism. A systematic review of management outcome studies. Thromb Haemost. 2009;101(5):886‐892. 19404542

[9] Perrier A Roy PM Aujesky D , et al. Diagnosing pulmonary embolism in outpatients with clinical assessment, D-dimer measurement, venous ultrasound, and helical computed tomography: a multicenter management study. Am J Med. 2004;116(5). doi:10.1016/j.amjmed.2003.09.041 14984813

[10] Wells PS Anderson DR Rodger M , et al. Excluding pulmonary embolism at the bedside without diagnostic imaging: management of patients with suspected pulmonary embolism presenting to the emergency department by using a simple clinical model and d-dimer. Ann Intern Med. 2001;135(2):98‐107. doi:10.7326/0003-4819-135-2-200107170-00010 11453709

[11] Lim W Le Gal G Bates SM , et al. American Society of Hematology 2018 guidelines for management of venous thromboembolism: diagnosis of venous thromboembolism. Blood Adv. 2018;2(22):3226‐3256. doi:10.1182/bloodadvances.2018024828 30482764 PMC6258916

[12] Sartori M Cosmi B Legnani C , et al. The Wells rule and D-dimer for the diagnosis of isolated distal deep vein thrombosis. J Thromb Haemost. 2012;10(11):2264‐2269. doi:10.1111/j.1538-7836.2012.04895.x 22906051

[13] Douma RA Mos ICM Erkens PMG , et al. Performance of 4 clinical decision rules in the diagnostic management of acute pulmonary embolism. Ann Intern Med. 2011;154(11):709‐718. doi:10.7326/0003-4819-154-11-201106070-00002 21646554

[14] Righini M Le Gal G De Lucia S , et al. Clinical usefulness of D-dimer testing in cancer patients with suspected pulmonary embolism. Thromb Haemost. 2006;95(4):715‐719. doi:10.1160/TH05-12-0791 16601844

[15] Cohen AT Agnelli G Anderson FA , et al. Venous thromboembolism (VTE) in Europe. The number of VTE events and associated morbidity and mortality. Thromb Haemost. 2007;98(4):756‐764. doi:10.1160/TH07-03-0212 17938798

[16] Evans TC Jehle D . The red blood cell distribution width. J Emerg Med. 1991;9(Suppl 1):71‐74. doi:10.1016/0736-4679(91)90592-4 1955687

[17] Felker GM Allen LA Pocock SJ , et al. Red cell distribution width as a novel prognostic marker in heart failure: data from the CHARM Program and the Duke Databank. J Am Coll Cardiol. 2007;50(1):40‐47. doi:10.1016/j.jacc.2007.02.067 17601544

[18] Montagnana M Cervellin G Meschi T Lippi G . The role of red blood cell distribution width in cardiovascular and thrombotic disorders. Clin Chem Lab Med. 2012;50(4). doi:10.1515/cclm.2011.831 22505527

[19] Tonelli M Sacks F Arnold M , et al. Relation between red blood cell distribution width and cardiovascular event rate in people with coronary disease. Circulation. 2008;117(2):163‐168. doi:10.1161/CIRCULATIONAHA.107.727545 18172029

[20] Lippi G Plebani M . Red blood cell distribution width (RDW) and human pathology. One size fits all. Clin Chem Lab Med. 2014;52(9). doi:10.1515/cclm-2014-0585 24945432

[21] Stubbs MJ Mouyis M Thomas M . Deep vein thrombosis. Br Med J. Published online February 22, 2018:360:k351. doi:10.1136/bmj.k351 29472180

[22] Konstantinides SV Meyer G Becattini C , et al. 2019 ESC guidelines for the diagnosis and management of acute pulmonary embolism developed in collaboration with the European Respiratory Society (ERS): the Task Force for the diagnosis and management of acute pulmonary embolism of the European Society of Cardiology (ESC). Eur Heart J. 2020;41(4):543‐603. 31504429 10.1093/eurheartj/ehz405

[23] Roy PM Douillet D Penaloza A . Contemporary management of acute pulmonary embolism. Trends Cardiovasc Med. 2022;32(5):259‐268. doi:10.1016/j.tcm.2021.06.002 34214598

[24] Lippi G Buonocore R Cervellin G . Value of red blood cell distribution width on emergency department admission in patients with venous thrombosis. Am J Cardiol. 2016;117(4):670‐675. doi:10.1016/j.amjcard.2015.11.024 26718227

[25] PatelGR MahapatraM AggarwalS SaxenaR . Serial values of hematologic variables and deep venous thrombosis: red cell distribution width is associated with deep venous thrombosis. Hematol Transfus Cell Ther. Published online December 5, 2022. doi:10.1016/j.htct.2022.11.00536481199

[26] Cay N Unal O Kartal MG Ozdemir M Tola M . Increased level of red blood cell distribution width is associated with deep venous thrombosis. Blood Coagul Fibrinolysis. 2013;24(7):727‐731. doi:10.1097/MBC.0b013e32836261fe 23751607

[27] Rezende SM Lijfering WM Rosendaal FR Cannegieter SC . Hematologic variables and venous thrombosis: red cell distribution width and blood monocyte count are associated with an increased risk. Haematologica. 2014;99(1):194‐200. doi:10.3324/haematol.2013.083840 23894011 PMC4007947

[28] Zöller B Melander O Svensson P Engström G . Red cell distribution width and risk for venous thromboembolism: a population-based cohort study. Thromb Res. 2014;133(3):334‐339. doi:10.1016/j.thromres.2013.12.013 24393657

[29] Bucciarelli P Maino A Felicetta I , et al. Association between red cell distribution width and risk of venous thromboembolism. Thromb Res. 2015;136(3):590‐594. doi:10.1016/j.thromres.2015.07.020 26220270

[30] Ellingsen TS Lappegård J Skjelbakken T Brækkan SK Hansen JB . Red cell distribution width is associated with incident venous thromboembolism (VTE) and case-fatality after VTE in a general population. Thromb Haemost. 2015;113(1):193‐200. doi:10.1160/TH14-04-0335 25274492

[31] Tunçez A Çetin MS Çetin EHÖ Yilmaz S Korkmaz A Uçar FM . Association between RDW and stent thrombosis in patients with ST-elevation myocardial infarction undergoing primary percutaneous coronary intervention. Medicine (Baltimore). 2017;96(5):e5986. doi:10.1097/MD.0000000000005986 28151892 PMC5293455

[32] Liang L Huang L Zhao X , et al. Prognostic value of RDW alone and in combination with NT-proBNP in patients with heart failure. Clin Cardiol. 2022;45(7):802‐813. doi:10.1002/clc.23850 35621296 PMC9286336

[33] Farah R Khamisy-Farah R . Significance of MPV, RDW with the presence and severity of metabolic syndrome. Exp Clin Endocrinol Diabetes Off J Ger Soc Endocrinol Ger Diabetes Assoc. 2015;123(9):567‐570. doi:10.1055/s-0035-1564072 26372846

[34] Rahimirad S Ghafari M Ansarin K Rashidi F Rahimi-Rad MH . Elevated red blood cell distribution width predicts mortality in acute exacerbation of COPD. Pneumol Buchar Rom. 2016;65(2):85‐89. 29542313

[35] Di Nisio M Van Es N Büller HR . Deep vein thrombosis and pulmonary embolism. Lancet. 2016;388(10063):3060‐3073. doi:10.1016/S0140-6736(16)30514-1 27375038

[36] Iorio A Kearon C Filippucci E , et al. Risk of recurrence after a first episode of symptomatic venous thromboembolism provoked by a transient risk factor: a systematic review. Arch Intern Med. 2010;170(19):1710‐1716. doi:10.1001/archinternmed.2010.367 20975016

[37] Boutitie F Pinede L Schulman S , et al. Influence of preceding length of anticoagulant treatment and initial presentation of venous thromboembolism on risk of recurrence after stopping treatment: analysis of individual participants’ data from seven trials. Br Med J. 2011;342:d3036. doi:10.1136/bmj.d3036 21610040 PMC3100759

[38] Schouten HJ Geersing GJ Koek HL , et al. Diagnostic accuracy of conventional or age adjusted D-dimer cut-off values in older patients with suspected venous thromboembolism: systematic review and meta-analysis. Br Med J. 2013;346:f2492. doi:10.1136/bmj.f2492 23645857 PMC3643284

[39] Weitz JI Fredenburgh JC Eikelboom JW . A test in context: D-dimer | Elsevier enhanced reader. J Am Coll Cardiol. 2017;70(19):2411‐2420. doi:10.1016/j.jacc.2017.09.024 29096812

[40] Crawford F Andras A Welch K Sheares K Keeling D Chappell FM . D-dimer test for excluding the diagnosis of pulmonary embolism. Cochrane Database Syst Rev. 2016;2016(8):CD010864. doi:10.1002/14651858.CD010864.pub2 27494075 PMC6457638

[41] Wu JX Qing JH Yao Y Chen DY Jiang Q . Performance of age-adjusted D-dimer values for predicting DVT before the knee and hip arthroplasty. J Orthop Surg Res. 2021;16:82. doi:10.1186/s13018-020-02172-w 33494760 PMC7831181

[42] Moynihan R Sanders S Michaleff ZA , et al. Impact of COVID-19 pandemic on utilisation of healthcare services: a systematic review. BMJ Open. 2021;11(3):e045343. doi:10.1136/bmjopen-2020-045343 PMC7969768 33727273

[43] Santana R Sousa JS Soares P Lopes S Boto P Rocha JV . The demand for hospital emergency services: trends during the first month of COVID-19 response. Port J Public Health. 2020;38(1):30‐36. doi:10.1159/000507764 PMC7206358 40504165

